# International clinical research networks – a collaborative approach for pandemic preparedness and response: The case of The Mexican Emerging Infectious Disease Clinical Research Network (LaRed)

**DOI:** 10.7189/jogh.13.03031

**Published:** 2023-07-14

**Authors:** Guillermo M Ruiz-Palacios, Justino Regalado-Pineda, Abelardo Montenegro-Liendo, Paola del C Guerra-de-Blas, Mary Smolskis, H Clifford Lane, The Mexican Emerging Infectious Disease Clinical Research Network (LaRed)

**Affiliations:** 1 Departamento de Infectología, Instituto Nacional de Ciencias Médicas y Nutrición Salvador Zubirán, Mexico City, Mexico; 2 Subdirección de Medicina, Instituto Nacional de Enfermedades Respiratorias Ismael Cosío Villegas, Mexico City, Mexico; 3 The Mexican Emerging Infectious Diseases Clinical Research Network (LaRed), Mexico City, Mexico; 4 Division of Clinical Research, National Institute of Allergy and Infectious Diseases, National Institutes of Health, Bethesda, Maryland, United States of America

In the last 100 years, the world has experienced four influenza pandemics, with an occurrence every 15-30 years resulting in an annual probability of 3% to 7% [1]. More recently, other pathogens including SARS, MERS, and the recent SARS CoV-2, have stressed the need to improve pandemic preparedness and response capabilities.

Developing strategies to promote robust basic and applied research with continuous monitoring of the human/animal interface to generate new knowledge applicable to public health policies and measures is critical to enhancing rapid responses to these outbreaks. Early detection and fast response to an epidemic or pandemic can be improved through an effective communication structure between countries worldwide. Such a structure should include strategically placed clinical research programmes and associated infrastructures.

## INTERNATIONAL CLINICAL RESEARCH NETWORKS – A COLLABORATIVE APPROACH FOR PANDEMIC PREPAREDNESS AND RESPONSE

The United States (US) National Institute of Allergy and Infectious Diseases (NIAID) supports collaborative, government-to-government research partnerships with the Ministries of Health (MoH) of Mexico, Indonesia, Mali, Guinea, Liberia, and the Democratic Republic of the Congo (DRC), designed to both improve long-term research capacity and promote rapid response to disease threats.

One key goal of these international collaborations is the establishment of human subjects’ research protocols to identify the early emergence of pathogens with pandemic potential. These collaborations also strive to determine the most effective treatments through randomised controlled trials (RCTs) designed and executed with adequate rigor to determine the safety and efficacy of potential therapies [2]. During the H1N1 pandemic, Tran et al. [3] highlighted that RCTs should be included at the forefront of public health response to pandemics. Similar recommendations were made by the US National Academies in their review of the research response to the 2014-2016 Ebola outbreak in West Africa [4].

The World Health Organization (WHO) has a research and development blueprint identifying diseases and pathogens that can cause a public health emergency due to their pandemic potential and/or a lack of effective treatments and vaccines. This list includes “Disease X”, representing the need to prepare for an unknown pathogen that could cause a significant public health emergency [5]. During the COVID-19 pandemic, RCTs evolved into platform trials to facilitate rapid evaluation of novel and re-purposed therapies. Platform trials, also referred to as master, basket, or umbrella trials, are a trial design that simultaneously tests multiple therapies, either individually or in combination, and multiple diseases in parallel, without having to develop individual protocols for every contingency [6].

The successful implementation of a clinical trial involves different actors, including representatives of affected communities, clinicians, biostatisticians, regulatory experts, laboratorians, pharmacists, regulatory experts, logisticians, and experts in communication. A clearly defined regulatory process, harmonised whenever possible on a global basis, and communication among regulators are crucial to their rapid implementation. International clinical research networks have substantially strengthened countries’ capabilities to implement clinical trials effectively and quickly.

Since the H1N1 pandemic, we have witnessed how pandemic response has been a model of ensuring the implementation of state-of-the-art science to inform and influence public health policy [3]. The recent COVID-19 clinical trials have highlighted the importance of collaborations; scientists sharing knowledge across public and private sectors led to the emergency authorisation/approval of COVID-19 vaccines in under than 10 months [7]. Long-term investments are required in both clinical trial networks and the studies themselves to be better prepared for emerging threats.

### The case of the Mexican Emerging Infectious Diseases Clinical Research Network (LaRed)

Successful evidence of the concepts mentioned above ie, surveillance, RCTs and regulatory communication is the The Mexican Emerging Infectious Diseases Clinical Research Network (LaRed), established in response to the 2009 H1N1 pandemic through a letter of intent signed between Mexican Minister of Health José Ángel Córdova and NIAID Director Anthony Fauci. The initial aim of LaRed was to develop a coordinated bi-national effort to conduct clinical research on influenza and potentially other infectious diseases. LaRed complements existing healthcare structures in Mexico and the US-generating scientific knowledge focused on treatment of emerging infectious diseases and respiratory viruses, with its mission being the generation of knowledge to improve health outcomes [8].

**Figure 1:**
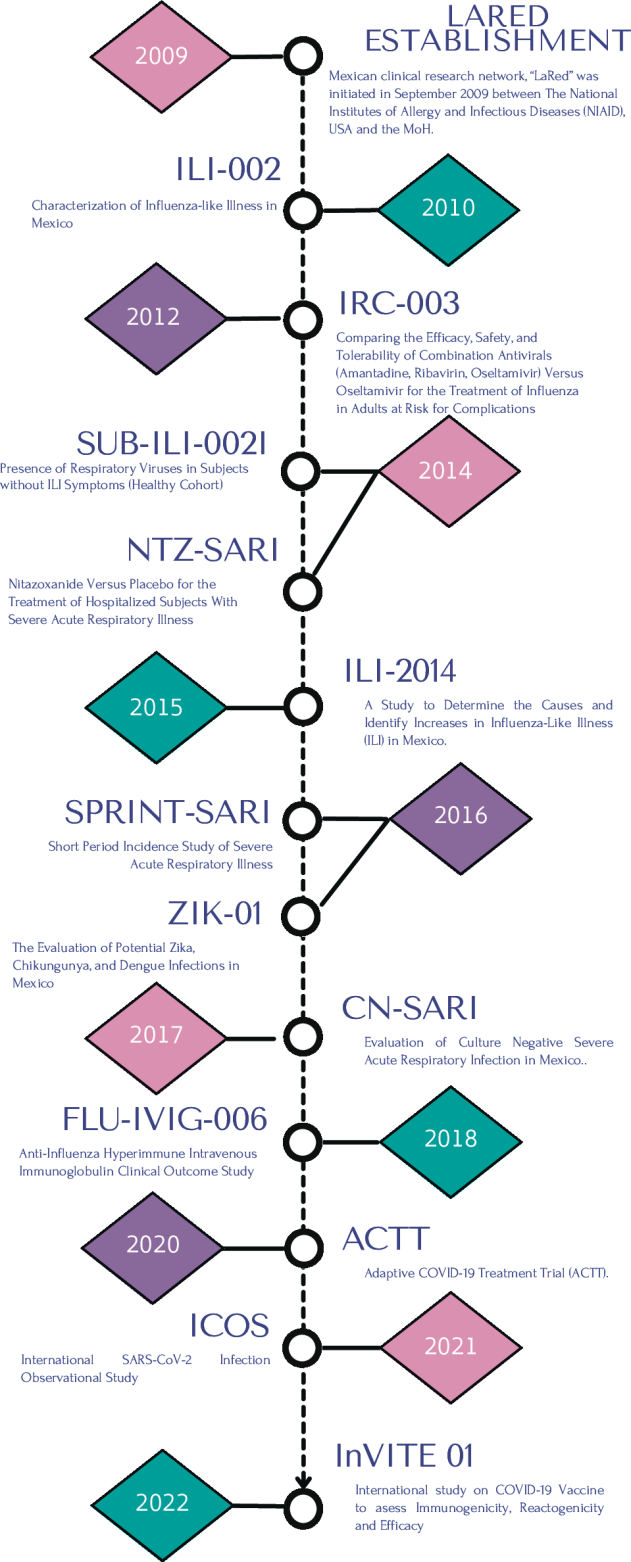
Studies conducted by LaRed. Since 2009, LaRed has conducted over 11 multicentre clinical studies and trials and is currently recruiting for two additional protocols.

Since 2009, LaRed has conducted over 11 multicentre clinical studies and trials and is currently recruiting for two additional protocols. It has enrolled over 8617 study subjects, focusing on pathogens with pandemic potential and other causes of febrile illnesses **(****Figure 1****)**.

Mexico is strategically located for the rapid identification of pathogens with pandemic potential. It has also become an immigration corridor for individuals from Africa, Asia, the Middle East, and Central and South America traveling to the North, who potentially bring new diseases that could pose a public health threat. Therefore, LaRed can rapidly mobilise in multiple clinical research sites in Mexico to implement surveillance, observational and clinical trials, as with influenza H1N1 pdm09, Zika, and COVID-19. Also, it has the capability for data collection on the natural history, vaccination, and breakthrough infections for various pathogens within crucial geographic regions.

**Figure:**
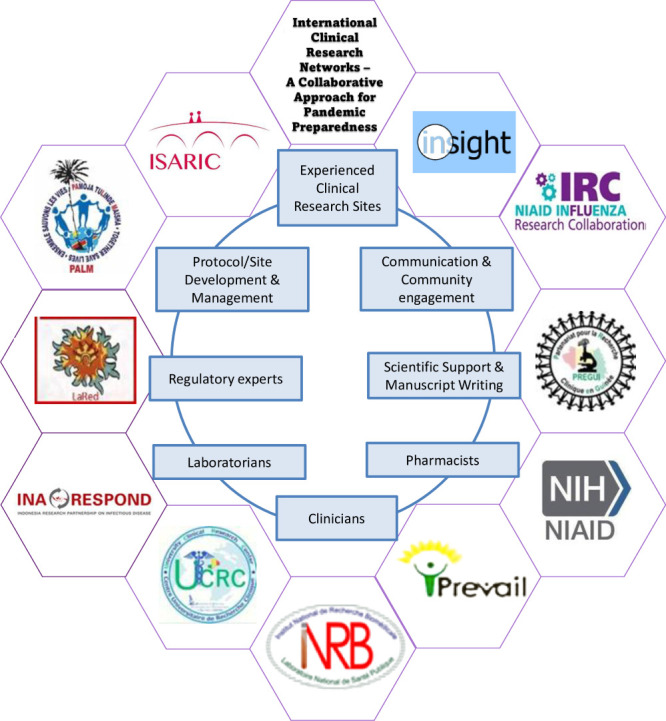
**Photo:** International clinical research networks ─ a collaborative approach for pandemic preparedness. The successful implementation of a clinical trial involves different actors, including representatives of the affected communities, clinicians, biostatisticians, regulatory experts, laboratorians, pharmacists, regulatory experts, logisticians, and experts in communication. International clinical research networks have substantially strengthened countries capabilities to implement clinical trials effectively and quickly. Source: Designed by Luis Mendoza-Garcés and Paola del Carmen Guerra-de-Blas. Each image segment is available for free use in the public domain.

During the COVID-19 pandemic, LaRed has implemented two observational studies (NCT04385251 and NCT05096091) and participated in two RCTs: an interventional study for the treatment of outpatients in the early stages of COVID-19 (NCT04910269) and the Adaptive COVID-19 Treatment Trial (ACTT) (NCT04280705). ACTT generated scientific knowledge on the use of potential treatments and allowed some subjects to benefit from treatments not originally available as standard of care in Mexico in the early phases of the pandemic. The platform design of ACTT allowed four different drugs [9-12] to be evaluated and led to the United States Food and Drug Administration approval of remdesivir and baricitinib in the treatment of COVID-19. This study contributed to the approval of remdesivir in Mexico and its acceptance and availability by the Mexico Ministry of Health in tertiary care COVID-19 hospitals and demonstrated how established international research networks can help deliver high-quality and productive research.

LaRed has also simultaneously collaborated with other networks across different continents. The group of six networks, alongside the ONUM (Sanskrit for spreading knowledge) Foundation in Mongolia, is conducting an observational cohort study of COVID-19 vaccine immunogenicity and allows the comparison of responses to different vaccines in different environments [13].

### Outstanding efforts made by other networks

Another example of how collaborative, government-to-government research partnerships have provided outstanding support to pandemic response is the *Pamoja Tulinde Maisha* (*PALM*) (Together Save Lives” in the Kiswahili language) Consortium at the Democratic Republic of the Congo (DRC), which operated through a multi-partner governance structure coordinated by WHO, the Ministry of Health, the Alliance for International Medical Action (ALIMA), and Médecins Sans Frontières (MSF) with the Institut National de Recherche Biomédicale and NIAID. They launched a RCT testing multiple investigational Ebola therapies, demonstrating that even during a conflict, clinical research can be conducted in an ethical, scientifically sound manner, and can help inform the outbreak response [14], contributing to the FDA approval use of mAb114 (Ebanga).

Other host country – US partnerships have been developed, usually established during an outbreak and maintained within efforts directed toward preparedness. These include the Partnership for Research on Ebola Vaccines in Liberia (PREVAIL), the Partnership of Clinical Research in Guinea (PREGUI), and the aforementioned *PALM* consortium (which were key in the detection and implementation of treatment protocols against Ebola), and the Indonesia Research Partnership on Infectious Diseases (INA-RESPOND) in Indonesia and the University of Clinical Research Center (UCRC) in Mali, studying causes of acute febrile illness, respiratory diseases, and tuberculosis, a critical re-emerging infectious disease. During the COVID-19 pandemic, clinical research programs in these countries were in place, acting as an essential structure to facilitate the needed rapid initiation of research protocols.

### Benefits from international clinical research collaborations

International research networks are a potent tool for delivering high-quality and productive research, encouraging co-operation and harmonisation to boost research delivery efficiency and facilitate preparedness to conduct clinical trials for health emergencies.

The cooperation and collaboration with the regulatory entities in these countries have led to effective clinical research efforts that have generated scientific knowledge, supporting prompt and inclusive public health decisions with a social perspective. For example, during the COVID-19 pandemic, regulatory entities around the global developed specific policy responses, including shortened administrative procedures and new forms of coordinating committees [15]. On November 2021, Mexico made a pivotal step in strengthening the regulation of pharmaceutical products and clinical research throughout the region, becoming the first Spanish-speaking country member of the International Council of Harmonization (ICH) in the Americas. In Mexico, regulatory entities are committed to provide regulatory certainty to achieve administrative simplification without deregulation, always following Good Regulatory Practices and Good Clinical Practices (ICH-GCP).

## CONCLUSION

The world must continue to support efforts on scientific research to prevent and/or effectively manage future pandemics caused by emerging infectious diseases. The “restless tide” of the microbial world, coupled with enhanced human mobility and environmental changes, necessitates a continued set of challenges from infectious diseases. Research partnerships and International Clinical Research Networks such as LaRed are essential to enhancing the ability of the global community to respond to the threat of emerging infectious diseases and to foster health diplomacy.

## References

[1] Nickol ME Kidrachuk J A year of terror and a century of reflection: perspectives on the great influenza pandemic of 1918-1919. BMC Infect Dis. 2019;19:117. 10.1186/s12879-019-3750-8 30727970 PMC6364422

[2] Sullivan GM Getting off the “gold standard”: randomized controlled trials and education research. J Grad Med Educ. 2011;3:285-9. 10.4300/JGME-D-11-00147.1 22942950 PMC3179209

[3] Tran TH Ruiz-Palacios GM Hayden FG Farrar J Patient-oriented pandemic influenza research. Lancet. 2009;373:2085-6. 10.1016/S0140-6736(09)61131-4 19541022

[4] Largent EA EBOLA and FDA: reviewing the response to the 2014 outbreak, to find lessons for the future. J Law Biosci. 2016;3:489-537. 10.1093/jlb/lsw046 28852537 PMC5570698

[5] World Health Organization. Prioritizing Diseases for Research and Development in Emergency Contexts. Available: https://www.who.int/activities/prioritizing-diseases-for-research-and-development-in-emergency-contexts. Accessed: 6 October 2022.

[6] Lu CC Li XN Broglio K Bycott P Jiang Q Li X Practical Considerations and Recommendations for Master Protocol Framework: Basket, Umbrella and Platform Trials. Ther Innov Regul Sci. 2021;55:1145-54. 10.1007/s43441-021-00315-7 34160785 PMC8220876

[7] Kalinke U Barouch DH Rizzi R Lagkadinou E Türeci Ö Pather S Clinical development and approval of COVID-19 vaccines. Expert Rev Vaccines. 2022;21:609-19. 10.1080/14760584.2022.2042257 35157542 PMC8935460

[8] Mexican Emerging Infectious Disease Clinical Research Netwrok (LaRed) 2011. https://www.redmexei.mx. Accessed: 6 November 2022.

[9] Beigel JH Tomashek KM Dodd LE Mehta AK Zingman BS Kalil AC Remdesivir for the Treatment of Covid-19 - Final Report. N Engl J Med. 2020;383:1813-26. 10.1056/NEJMoa2007764 32445440 PMC7262788

[10] Kalil AC Patterson TF Mehta AK Tomashek KM Wolfe CR Ghazaryan V Baricitinib plus Remdesivir for Hospitalized Adults with Covid-19. N Engl J Med. 2021;384:795-807. 10.1056/NEJMoa2031994 33306283 PMC7745180

[11] Wolfe CR Tomashek KM Patterson TF Gomez CA Marconi VC Jain MK Baricitinib versus dexamethasone for adults hospitalised with COVID-19 (ACTT-4): a randomised, double-blind, double placebo-controlled trial. Lancet Respir Med. 2022;10:888-99. 10.1016/S2213-2600(22)00088-1 35617986 PMC9126560

[12] Kalil AC Mehta AK Patterson TF Erdmann N Gomez CA Jain MK Efficacy of interferon beta-1a plus remdesivir compared with remdesivir alone in hospitalised adults with COVID-19: a double-bind, randomised, placebo-controlled, phase 3 trial. Lancet Respir Med. 2021;9:1365-76. 10.1016/S2213-2600(21)00384-2 34672949 PMC8523116

[13] Sereti I Shaw-Saliba K Dodd LE Dewar RL Laverdure S Brown S Design of an observational multi-country cohort study to assess immunogenicity of multiple vaccine platforms (InVITE). PLoS One. 2022;17:e0273914. 10.1371/journal.pone.0273914 36107966 PMC9477293

[14] Mulangu S Dodd LE Davey RT Jr Tshiani Mbaya O Proschan M Mukadi D A Randomized, Controlled Trial of Ebola Virus Disease Therapeutics. N Engl J Med. 2019;381:2293-303. 10.1056/NEJMoa1910993 31774950 PMC10680050

[15] Organization for Economic Co-operation and Development. Policy Responses to Coronavirus (COVID-19. Regulatory quality and COVID-19: The use of regulatory management tools in a time of crisis. 2020. Available: https://www.oecd.org/coronavirus/policy-responses/regulatory-quality-and-covid-19-the-use-of-regulatory-management-tools-in-a-time-of-crisis-b876d5dc/#biblio-d1e1887. Accessed: 6 October 2022.

